# Canadol, a New Local Anæsthetic

**Published:** 1888-02

**Authors:** 


Canadol, a New Local Anæsthetic.—Canadol is a hydrocarbon derived from petroleum. It is a light, volatile, exceedingly inflammable liquid, with an odor resembling that of benzine. It is not soluble in either water or alcohol. By its rapid evaporation, it will lower temperature more rapidly than ether. Canadol may be used in slight operations in the same manner as the ether spray.—*L' Oculistique.*

